# Supplementary material for: Are carbon emissions trading and green financial instruments synergistic? -Comprehensive quantitative research based on content analysis
Source: PLoS One. 2024 Mar 7;19(3):e0298601. doi: 10.1371/journal.pone.0298601 (PMC10919601; doi:10.1371/journal.pone.0298601)
Supplement: S1 Data — (ZIP) [file pone.0298601.s001.zip › Data/地级市Z_F工作报告.zip!/─■╧─/╩»╫∞╔╜╩╨/└┤╫╘╣1⁄2╓┌║┼í╛┬φ┐╦╩2╛▌═°í┐_2015.docx]

各位代表：

现在，我代表市人民政府向大会作工作报告，请予审议，并请市政协委员和其他列席人员提出意见。

一、2014年工作回顾

2014年，是我市经济发展极为困难的一年。面对宏观经济形势更趋严峻复杂，经济下行压力加大等一系列前所未有的考验，我们全面贯彻落实自治区党委、政府和市委的决策部署，围绕市第十三届人大二次会议确定的目标任务，攻坚克难，综合施策，打赢了保增长攻坚战，经济社会发展稳中有进、稳中向好。全年实现地区生产总值467.2亿元，增长6.7%；完成固定资产投资498亿元，增长8.5%；完成地方公共财政预算收入29.1亿元，可比增长15%；城镇居民和农村常住居民人均可支配收入分别达到22380元、10215元，分别增长8.1%和10.1%；城镇登记失业率为3.8%。

(一)全力以赴保增长，工业经济逆势回升。应对工业经济快速下行局面，强化措施，全面打响保增长攻坚战。实行市级领导和县区、园区负责人帮扶停产、减产企业责任制，成立8个专项推进组，一企一策帮助解决难题。积极争取自治区直供电、差别电价等政策支持，及时出台稳定工业增长意见，对重点项目投产达效、企业复产增产等给予扶持，兑现各级、各类帮扶资金9.95亿元。全市新增规模以上企业30家，规模以上工业实现增加值225.5亿元，增长7.5%。强化金融风险防控，出台金融支持企业30条，部分企业信用风险得到逐步化解和控制。强化信贷支持，市域银行机构全年新增贷款27.7亿元，各项贷款余额418.6亿元 侯卫东官场笔记。加强融资平台建设，组建成立鑫鼎担保公司。推进企业直接融资，5家企业在新三板、天交所挂牌交易，晟晏集团、大地循环、吉元冶金等企业通过发行企业债、短期融资券等方式，实现债权融资24.7亿元。

(二)下大力气抓项目，投资实现稳步增长。出台重大项目推进管理办法，建立加强改进政府投资项目管理制度，实行重大项目审批全程代办、限时办结、并联审批和考核奖励机制。申银特钢、丽珠药业、维尔铸造、国际建材城等项目投产达效，中色东方铍材加工、大地轮胎、金晶玻璃、力帆凯马等项目超额完成年度投资，快速推进中利腾晖光伏电站等项目。修订完善了招商引资优惠政策，改进项目认定和考核奖励办法，招商到位资金345.9亿元；争取各类资金72.83亿元，可比增长15.2%。我市被列为国家信息惠民城市试点、全国资源综合利用“双百工程”示范基地、“宁夏银川—石嘴山承接产业转移示范区”，生态经济开发区被列入全国循环化改造试点，与天津滨海新区缔结为友好市区。

(三)积极主动调结构，产业转型有序推进。制定实施产业转型升级和结构调整“1+3”方案、老工业基地调整改造规划，加快产业转型升级。16个亿元以上配套协作项目、13家企业炉型置换和烟气余热综合利用项目有序推进，建成投产工业项目109个，淘汰落后产能61万吨，促进了工业提质增效。加快陆港口岸便利通关模式构建，实现属地申报、口岸验放，申银铁路物流园等项目取得积极进展。环湖开发实施文化旅游和基础设施项目13个，完成投资4.58亿元。中华奇石文化城、北武当生态旅游区、五七干校原址建设等项目进展顺利。与农垦集团签订战略合作协议，促进垦地融合。推进电子商务发展，2家电子商务创业孵化园和“宁夏优品”电商平台正式运营。现代农业加快发展，国家农业科技园区建设稳步推进，完成投资3.12亿元。组织实施百万亩盐碱地改良工程，改造中低产田27.3万亩，新增节水灌溉面积16.4万亩，农业综合生产能力得到提升。粮食总产量达到40.9万吨，实现“十一连增”。特色种养殖业发展势头良好，农产品加工转化率达到60%，农业保障体系不断加强。坚持创新驱动发展，加快孵化园、企业研发中心等创新平台建设，推动科技创新成果转化，科技对经济增长的贡献率达到45.7%。注重市场主体培育和品牌建设，全年新增企业1593户，个体工商户7864户，荣获中国驰名商标2个，宁夏著名商标20个。

(四)多措并举抓统筹，城乡建设取得突破。举全市之力冲刺全国文明城市创建，紧紧围绕“八大环境一个创建”总目标，扎实开展公共环境整治、公共秩序整治和公共关系建设，推进违法占地违法建设行为专项整治，创建体制机制进一步完善，群众参与热情空前高涨，在自治区开展的创城满意度调查中，我市群众满意度达到98%。重新调整全市镇村体系规划，完成14个美丽小城镇(村庄)规划建设。进一步完善大武口南片区医院、学校、商贸等公共服务设施配套，市儿童医院暨妇幼保健院、银城建材装饰城、舍予园市场等项目快速推进。石嘴山至银川同城化公路建成通车，石嘴山黄河公路大桥改扩建、简礼公路等重点交通项目加快实施，完成70个公交站点建设。开通银川至石嘴山城际列车，新增市区至火车站等4条公交线路，方便了群众出行。主干道路大整治大绿化、贺兰山东麓生态防护林、水生态建设等重点工程加快推进，完成造林绿化8万亩，被确定为全国水生态文明城市建设试点。推进煤炭集中区环境综合整治，取缔、搬迁涉煤企业67家，完成规范发展煤炭市场攻坚目标。积极开展“国土资源执法年”活动，严厉打击煤炭、砂石盗采行为。启动环境保护行动计划、大气污染防治三年行动计划，实施了一批污染治理项目，环境质量进一步改善。

(五)加大投入惠民生，社会事业不断繁荣。将77.5%的财力用于保障和改善民生，十件民生实事顺利实施。棚户区改造加快推进，完成投资23亿元，新建续建住宅19519套，建成7193套。投资1.9亿元，建设公共租赁(廉租)住房2750套，建成1917套，实行廉租住房、公共租赁住房并轨管理。完成老旧小区节能改造26万平方米，住房公积金归集使用更加惠民。扎实做好移民工作，建成移民住房1833套，开展移民就业培训3300人次，实现就业12775人。建成1000平方米大学生创业孵化园，带动106名大学生实现创业。积极培育新型职业农民，被列入国家新型职业农民培育工程示范市。坚持教育优先发展，实施“两名三提高”工程，加快“校校通、班班通暨数字化校园”建设，被评为全国教育优先发展示范城市，在全区率先实现义务教育基本均衡发展。强化基本公共卫生服务，13家公立医院启动“先住院、后付费”诊疗服务，被评为全国无偿献血先进城市，3家医院被评为全国百姓放心示范医院，平罗县被确定为全国县级公立医院综合改革试点。依法平稳实施“单独两孩”政策。积极推进国家公共文化服务体系示范区创建工作，群众性文化活动蓬勃开展，成功举办国际铁人三项赛、全国室内五人制足球赛和全国户外运动嘉年华暨露营大赛。积极争取自治区减免15家困难企业职工社会保险滞纳金222万元，争取职工养老保险调剂金6.3亿元，清理养老保险历史欠费4667万元；安排7400多万元对133家困难企业进行社保补贴、岗位补贴、技能培训补贴；开展城乡居民大病保险试点工作。关爱老年人和困难群众，为2500户老年人家庭安装“一键式”呼叫器，为4.1万困难群众发放低保金9024万元，建设慈善超市10家。积极创建全国法治城市，办结法律援助诉讼案件869件，为困难群众挽回经济损失2383万元。积极开展民族团结创建和宗教场所安全隐患排查活动，强化食品安全专项整治。深入推进百日安全大排查大整治，严格落实企业安全生产主体责任。加大信访责任管理目标考核和重点信访事项督办力度，大力推进平安石嘴山建设，荣获全国社会管理综合治理先进集体。加强防震减灾工作，荣获全国防震减灾工作综合考核先进单位。

(六)坚定不移促改革，发展活力明显增强。加快推进重点改革落地，启动了政府机构改革，顺利完成工商、质检系统划转接收，机构设置更加科学，运行机制更加完善。推进行政审批制度改革，市本级行政审批事项由230项调减为67项，减少71%，承接自治区政府下放的86项行政审批事项调整为31项，平均办结时限压缩52%；取消和减免市本级65项行政事业收费。加快国企改革，推进重组整合，市属国有企业由20户减少到8户，基本实现政企分离。实施星瀚集团燃气公司混合所有制改革，国企改革取得较大突破。继续推进平罗县国家农村土地经营制度改革试验区建设，开展农村集体“三资”管理制度改革、农民土地经营权入股和以地养老等试点，积极推进“三权”抵押，共办理抵押5100多笔，贷款2.8亿元，农村土地改革经验在全国交流。

(七)持之以恒转作风，政府效能不断提升。扎实开展党的群众路线教育实践活动，坚持边查边改，狠抓专项整治，解决了一批突出问题，促进了作风转变和效能提升。削减“文山会海”，各类会议、文件同比分别减少20%和15%。严格审批各类活动，清理创评活动32项，取消社区开具证明事项39项，撤销、整合议事协调机构237个，精简率达81%。加强政府部门平时监督，实行按季考评，邀请人大代表、政协委员和服务对象进行满意度测评。强化政务督查和效能监察，建立完善决策执行机制和重大事项督查督办机制。开辟电视访谈节目，30个政府部门负责人对重点工作公开承诺，接受社会监督。举办“电视问政”4期，曝光了一批违反作风建设规定的典型问题，达到了规范工作、激发动力、提高效率的目的。畅通网络问政渠道，59个部门开通政务微博，石嘴山网访问量突破一千万人次。强化审计监督，核减工程价款4689万元，追缴各类财政资金1.16亿元。规范公务用车配备，收回公务用车81辆。严格执行办公用房标准，清理超标办公用房15000平方米。强化“三公”经费动态监管，部门预决算和“三公”经费预算全部公开，市级党政机关行政运行支出同比下降10.5%。自觉接受人大、政协、人民群众和新闻媒体的监督，办理人大代表议案、建议62件、政协委员提案178件，办复率达到100%。

一年来，物价、统计、市场监管、人防、供销、气象、扶贫助残、国防动员等工作都取得了新成绩，全市获得省部级以上各类表彰奖励29项。

各位代表，过去的一年，我们勇担重任、敢于担当、奋力攻坚，大力推进重点工作、重大项目和重点改革，着力改善民生，做了大量卓有成效的工作。可以说，挑战非同寻常，过程充满艰辛，成绩来之不易。这是市委把握大局、正确领导的结果，是市人大、政协加强监督、鼎力支持的结果，是各县区、各部门同舟共济、攻坚克难的结果，更是全市人民不懈努力、共同奋斗的结果。在此，我代表市人民政府，向全市广大干部群众、驻石部队指战员、武警消防官兵、公安民警致以崇高的敬意!向大力支持政府工作的各位人大代表、政协委员，向各民主党派、工商联、人民团体及社会各界人士表示衷心的感谢!

在总结成绩的同时，我们更加清醒地认识到，全市经济社会发展还面临不少困难和问题，主要表现在：宏观经济形势仍然严峻复杂，经济下行压力持续加大，经济结构和产业发展积累的问题难以在短时间内解决，推动产业转型升级任重道远；支撑转型发展的大项目少，特别是产业项目储备不足，固定资产投资乏力；受税制改革、经济下行和政策性减收等因素影响，财政增收困难，收支矛盾突出；民生呼声和期望越来越高，保障和改善民生的任务艰巨；一些干部作风不实，担当意识、服务意识、勤政廉洁意识不强，四风方面的突出问题还不同程度地存在。对此，我们将高度重视，正视问题，切实加以解决。二、2015年的工作

2015年是全面落实“十二五”规划的收官之年，也是我市稳定经济增长、推进产业转型升级的关键之年，做好今年的各项工作意义重大。当前，世界经济仍处在深度调整期，总体复苏疲弱态势难有明显改观。我国经济步入以中高速、优结构、新动力、多挑战为主要特征的新常态，经济运行中的问题和矛盾相互交织，下行压力依然较大。我区经济发展外部条件也发生着新变化，依靠资源和环境投入支撑的高增长已难以为继。我市正处于转型发展的关键时期，面临着保增长与保节能的双重压力，面临着依靠投资拉动增长与项目严重不足的双重矛盾，面临着财政增收困难与民生刚性需求增加的双重考验，面临着转型升级迫切与企业技改投资能力不足、招商引资难度加大的双重难题。面对严峻形势，我们必须以更加强烈的紧迫感和使命感，迎难而上，敢打硬仗，坚定不移地推进工业强市，坚定不移地推进“三大转型”，坚定不移地推进深化改革和依法治市，坚定不移地弘扬“五湖四海、自强不息”的石嘴山精神，积极主动应对，全力破解难题，着力提高经济发展质量和效益。从有利条件看，我国经济发展总体向好的基本面没有改变，重点领域和关键环节改革正加快落地，“一带一路”建设全面启动，内陆开放型经济试验区建设加快推进，中阿博览会影响不断扩大，东部产业转移和老工业基地调整改造等逐步实施，这些都为石嘴山的发展带来了新机遇。更重要的是，我们有市委的坚强领导，有市人大、政协的大力支持，有战胜困难的宝贵经验，有创新突破的务实举措，有敢打硬仗能打胜仗的干部队伍，有全市上下的负重拼博，这些都为我们推动发展提供了有力保障。面对新的形势，我们必须进一步强化责任意识、担当意识，振奋精神、攻坚克难，坚持底线思维和问题导向，正确认识新常态，主动适应新常态，积极把握新常态，坚定信心，务实苦干，全力以赴稳增长、促改革、调结构、惠民生、防风险，促进全市经济平稳健康发展和社会和谐稳定。

今年政府工作的总体要求是：认真贯彻落实党的十八大和十八届三中、四中全会及习近平总书记系列重要讲话精神，按照自治区党委十一届四次、五次全会和市委九届五次全会部署要求，主动适应经济发展新常态，坚持稳中求进工作总基调，把产业转型升级和结构调整、提高经济发展质量和效益作为主攻方向，加快推动产业、民生、生态“三大转型”，继续深化改革，推进依法治市，全面加强政府自身建设，为建设开放富裕和谐美丽新型工业城市、在全区率先全面建成小康社会奠定更加坚实的基础。

主要奋斗目标是：地区生产总值增长7%左右，全社会固定资产投资增长10%，地方公共财政预算收入可比增长6%，社会消费品零售总额增长8%，城镇居民和农村常住居民人均可支配收入分别增长8.5%、9.5%，城镇登记失业率控制在4%以内，完成自治区下达的年度节能减排任务。

完成上述目标，需要突出抓好以下工作：

(一)加快产业转型，提质增效调结构

正确认识新常态，把发展质量和效益放在首位，抓住石嘴山国家级资源枯竭型城市转型示范区列入自治区转型升级方案十大任务契机，落实老工业基地调整改造规划，建立统计监测评价考核体系，推进产业转型升级和结构调整“1+3”方案实施。

提高工业发展质量效益。坚持有保有压，做好传统产业优化存量、新兴产业培育增量、节能减排淘汰减量三篇文章，实施“4344”重大任务，培育壮大新材料、装备制造、电石化工和冶金四大产业集群，加快发展新能源、生物医药、新型煤化工三个特色产业，实施节能降耗、治污减排、工业节水、化解和淘汰落后产能四大专项行动，打造四大园区转型平台，推动工业转型升级和结构调整。深入实施“双龙工程”，有计划地在每个集群和特色产业上培育1—3家龙头企业，重点培育大地循环、申银特钢2家销售收入过百亿元企业和中色东方、福华冶金、吉元冶金3家50亿元企业。鼓励大型企业通过服务外包、订单生产等方式，带动中小微企业发展配套产品，培育发展维尔铸造、银晨太阳能、恒通管业、科捷锂电池等一批科技型中小企业。加快轻工业发展，提升占比1个百分点。大力实施创新驱动战略，引导和支持有条件的企业建立技术开发中心、组建产业技术创新联盟，开展关键技术攻关和新产品开发，加速创新成果产业化。促进信息化和工业化深度融合，运用高新技术和先进适用技术，改造提升传统产业，实施神华宁煤炭基公司热动力站、惠冶镁业镁合金深加工、日盛实业热电联产等50个技改项目，重点抓好吉鑫冶金、惠义冶金等5家高载能企业炉型置换、搬迁改造和烟气余热综合利用。加快实施石嘴山经济技术开发区、生态经济开发区43个循环化改造项目，推进资源综合利用“双百工程”示范基地建设，资源综合利用率提高3个百分点。用足、用活、用好老工业基地调整改造、承接产业转移示范区、国家级开发区政策，实施开发区扩区调位，推进国家级开发区提升发展，加强专业园区建设，支持有条件的园区开展水、电、汽综合利用和工业污水集中处理及回用，建设转型试点园区。投资6.2亿元，完善园区道路、供排水等基础设施，着力改善投资环境，提升开发区承载力。

转变农业发展方式。以国家农业科技园区建设为抓手，按照产出高效、产品安全、资源集约、环境友好的要求，着力打造“一岸三路”产业带，建设“一园四区”产业园，发展壮大“一优五特”产业。发挥上海种业、宁羊农牧、富民水产、华泰农科技等龙头企业带动作用，建设制种产业示范区13万亩，清真肉羊标准化、规模化养殖场30个，生态水产、露地瓜菜示范区各6万亩，特色优势产业产值占农业总产值86%以上，农产品加工转化率达到63%。加快农业科技成果转化，提高良种覆盖率，示范推广育繁杂交改良、水肥一体化、机械化育苗等先进适用技术。挖掘粮食增收潜力，创建粮食高产示范区10个，优质粮食种植面积达到46万亩以上。加强农技服务体系和流通体系建设，积极开拓农产品市场，在北京、上海等城市设立农产品销售窗口。开展全国新型职业农民培训试点，年内新培育家庭农场、专业合作社等各类经营主体500家，总数达3100家，带动农户5.5万户。全面建成平罗县农村土地经营制度改革试验区，完成土地确权登记颁证工作，建设全国农村土地改革先行区。推进银北地区百万亩盐碱地改良工程，整合农业综合开发、国土整治等项目，治理五一支沟、五三支沟等骨干沟道35条，推进高效节水示范项目建设，改造中低产田20万亩，建设高标准农田10万亩，新增节水灌溉面积12万亩，夯实农业稳定发展基础。

推动三产扩张提升。突出陆港与沙湖的带动作用，提升以现代物流业为重点的生产性服务业，扩张以文化旅游业为重点的生活性服务业，统筹发展金融、电子商务等现代服务业，力争第三产业比重提高1个百分点。加快惠农陆港电子口岸建设，力促宁津物流园全面开工，加快富海物流园二期、西部吉运国际物流产业园、宁夏邦德物流园建设，启动国家外贸转型升级专业化工品示范基地培育。积极发展文化旅游业，深化与沙湖在旅游规划上的对接融合。推进星海湖国家级文化产业试验园区建设，完善环星海湖区域基础设施，加快环湖岸线整治和水系景观工程建设，加快推进星海银河景区、中华奇石文化城等项目。立足房地产市场现状，完善医院、学校、市场等配套公共基础设施，举办房车文化生活博览会，抓好商品房促销，推行政府回购商品房用于保障性住房新模式，探索利用开发商闲置土地建设廉租住房，激活房地产市场。做好专业街区规划，加快汉唐商业街、石嘴山综合商贸城、万宇商业广场等特色商业街区建设。推进金融IC卡应用，实现在公交、出租等行业的快速支付。

(二)坚持综合施策，精准发力保增长

主动适应新常态，理性面对增速放缓，沉着应对下行压力，总结过去协调运行的好经验，创新方式和手段，着力解决经济运行关键问题，确保经济增长在合理区间。

灵活调控运行。密切关注产业发展外部形势和重点工业品市场动态，紧盯增长与节能的趋势性变化，逐月逐季分析经济运行，及时调整调控政策，增强运行协调的预见性、灵活性、针对性和精准度。全面梳理增量因素，抓牢确定的工业增长点，力促已建成的大地轮胎、申银特钢、福华冶金等项目充分释放产能，形成增量88亿元。抑制减量影响，加强对有色金属、煤矿机械行业支持，控制减量在18亿元以内，确保全市规模以上工业增加值增长8%。强化增收节支，积极化解实施资源税改革和取消太西煤价格调节基金对财政收支带来的巨大影响，大力培植财源，优化支出结构，提高财政管理绩效和资金使用效益，确保不出现政府债务逾期。

加强企业服务。继续实施市级、县区领导包推规模以上工业企业、准规上企业责任制和派驻企业指导员等制度，加大对非公企业服务力度，健全上门服务、跟踪帮扶长效机制，指导企业树立战略思维、完善发展规划、创新经营管理、加快技改升级，帮助解决项目审批、企业融资等实际困难和问题。有针对性地开展困难企业帮扶，帮助符合产业政策的企业尽快摆脱困境，确保工业企业停产面控制在15%以内，亏损面收缩到50%以内。加强企业家队伍建设，继续办好企业董事长(总经理)培训班，加大培训力度，提升经营管理水平。

强化金融支撑。高度重视金融风险防控，落实金融支持企业保增长促发展的意见，坚持“政府帮扶、市场主导、一企一策、联手行动”的处置原则，落实县区主体职责，严守不发生区域性、系统性金融风险的底线。完善全市中小企业信用档案，确定重点支持企业和淘汰落后企业名单。加强小额贷款公司等非存款类融资机构监管，规范金融秩序。积极搭建银企合作平台，充分发挥鑫鼎担保公司作用，引导各银行机构合理投放信贷。加快推进直接融资，推动企业在新三板、天交所等挂牌交易，鼓励引导发行中小企业私募债、区域集优债、短期融资券等，帮助企业拓宽融资渠道，调整融资结构。

差别政策扶持。加强政策研究，对企业实行差异化扶持政策，集中财力支持发展前景好、带动能力强、转型调整快的企业，提高财政资金使用效力。落实《强化政策支持加快推进转型发展的若干意见》，探索建立产业转型升级引导基金，对企业科技创新、技改升级等予以重点扶持。积极争取国家和自治区的政策倾斜，对鼓励发展的优势和特色项目给予贷款贴息，优先申报国家和自治区专项资金。继续落实扩大差别电价、增加直接交易电量、产需直供企业补贴、高载能企业搬迁改造等优惠政策，争取援企稳岗政策，支持企业恢复扩大生产。

(三)力推项目建设，千方百计增投资

积极把握新常态，培育经济发展持久动力，发挥投资对经济发展的关键作用，抓住产业项目这一核心，努力扩大投资规模，优化投资结构，增强投资拉力。

提速重点项目建设。以产业项目为重点，抓紧实施一批投资规模大、带动力强的大项目，确保形成更多的投资，以重点项目支撑投资稳定增长。突出抓好122个重点项目建设，完成投资219亿元以上。工业方面，重点实施大唐国际平罗火电、国电大武口热电联产、中利腾晖线缆、金晶玻璃、大地轮胎等37个项目，完成投资119.4亿元；围绕工业骨干龙头企业，推进坤虹铜材深加工、华益冠益高分子PVC等35个亿元以上配套协作项目建设，促进工业投资持续增长；服务业方面，重点实施红星美凯龙城市综合体、德美斯物流园、万德隆商业广场、宁夏汇创农机农资城等28个项目，完成投资31亿元；农林水及生态建设方面，重点实施银北百万亩盐碱地改良、农垦简泉奶牛养殖、西海湖清真牛肉、第四排水沟治理、大武口森林公园改造等16个项目，完成投资14.5亿元；基础设施建设方面，重点实施红礼黄河公路大桥、市检验检测认证中心等27个项目，完成投资29亿元；民生社会事业方面，重点实施星海中学、职教中心三期、龙辰苑老年公寓等14个项目，完成投资25.2亿元。不断完善领导干部包推项目责任制，对重点项目实行“一套班子、一抓到底”的全程服务。

全力开展招商引资。把招商引资重点聚力到产业项目上，突出招大引强和集群配套，运用市场化模式完善基础设施配套，围绕产业链、设备链和资源链，以提升落地企业潜在竞争力为核心，吸引更多企业进行产业上下游配套。推动北京蓝星化工、中国化工装备集团与大地循环异氢酸酯、上海挪宝新能源地源热泵成套设备、神华集团30万辆电动摩托车等项目尽快落地。改进招商考核，突出产业投资，力争全年招商引资到位资金350亿元以上。增强争项目、争资金工作主动性，积极走出去、向上跑，争取更多的项目、资金及政策支持，全年争取资金增长10%。

精心谋划储备项目。坚持一手抓项目建设，一手抓项目储备，做到重大项目建设前后接续、梯次跟进。紧盯国家、自治区重点支持领域，围绕产业转型方向，精心谋划大项目，充实完善项目库，确保储备项目3000亿元以上。加强与国家、自治区有关部门的对接，谋划储备一批促转型、提质量、增效益的大项目，争取更多项目列入国家和自治区“十三五”规划。积极做好自治区60周年大庆重大项目申报工作，争取石嘴山沙湖支线机场、华电集团发电等列入自治区重点前期项目，尽快开工建设。

(四)推进新型城镇化，优化格局抓统筹

更加突出规划引领，以基础设施建设为重点，统筹城乡一体发展，积极稳妥推进以人为核心的新型城镇化建设。

优化城乡发展布局。认真贯彻落实宁夏空间发展战略规划与城镇体系规划纲要，构建科学规划编制体系，合理配置空间资源，优化城镇功能布局。按照大武口东拓南进、惠农北扩南连、平罗西拓南进的总体思路，推进石银一体化发展，促进老城区、新区、开发区协调推进。依托现有城镇发展基础，整合交通、资源等优势，高标准完成重点镇和中心村建设规划，提升美丽乡村规划品质。

完善城乡基础设施。开展城市供气、供热、供水管网普查，积极争取国家北方地区集中供热老旧管网改造和全国城镇燃气老旧管网改造项目，提升保障能力。加快城市免费公共卫生间、垃圾无害化处理等设施建设，推进老旧小区节能改造。提升美丽乡村建设水平，规划建设美丽小城镇2个、美丽村庄10个。完成石嘴山黄河公路大桥续建工程，开工建设大武口至平罗同城化连接线、五岳路、京藏高速公路(平罗)至西线高速连接线等工程，做好京藏高速公路石嘴山段改扩建工程前期工作。

提高城市管理水平。加快智慧城管平台建设，实现城市管理信息系统全市联网、统一管理。扎实开展建筑质量提升年和物业管理提升年活动，实施工程质量治理两年行动计划，着力打造精品工程。研究出台物业管理政策，提高物业企业管理服务水平。加大“两违”整治力度，坚决查处违法建设和违法占地，推进城乡结合部环境整治。

持续推进“六城联创”。巩固提高全国文明城市创建成果，深化各类文明创建活动，抓好公共环境和公共秩序整治，提高城市精细化管理水平，建立健全长效机制，不断提高市民素质和城市文明程度。加大“双拥”工作力度，推进军民共建、军地融合发展，争创全国双拥模范城。以创建全国文明城市为龙头，带动国家园林城市、卫生城市等“六城联创”，提升城市的内在品质和外在形象。

(五)着力改善生态，持之以恒优环境

坚持不懈改善生态环境，加大生态治理和环境保护力度，着力优化人居环境，提高经济竞争软实力。

强力推进节能减排。树立减量也是成绩的理念，在保增长的同时，更加注重政策灵活性，适时调整出台节能减排强制性约束政策，确保完成“十二五”节能降耗任务。加大落后产能淘汰力度，落实限制类和淘汰类企业差别电价政策，鼓励支持企业实施窑炉改造、余热余压利用、电力需求侧管理、能量系统优化、固废综合利用等节能示范工程。严格控制电石、铁合金等高载能企业产能总量，确保万元GDP能耗下降、能源消费总量完成自治区下达指标 完美世界小说。加大规模小、工艺差、污染重企业关停力度，鼓励支持企业技改升级，控制污染排放总量，促使落后产能逐步退出市场。加强城区扬尘防治，淘汰城区燃煤锅炉。

加大生态建设力度。围绕创建国家园林城市，着力推进造林绿化，完成林业生态建设4万亩。加快市民休闲森林公园建设，完成公园主要景点建设和70%的人工造林任务。巩固主干道路大整治大绿化成果，进一步提高城区、村庄绿化水平。实施归韭沟景观水系及柳条沟河道治理等工程，提高城乡防洪标准。抓好全国水生态文明城市试点工作，制定实施《水污染防治行动计划》，加快工业园区污水处理、中水回用等基础设施建设，在有条件的园区探索社会资本参与工业污水处理和固废处置。推进城市污水厂提标改造，强化对沙湖、星海湖等重点水域及水源地的保护，保障饮用水安全。加强大气污染防治，严禁焚烧农作物秸杆，加大生态恢复力度，努力控制面源污染。

巩固规范发展煤炭市场成果。继续推进煤炭集中区治理，加强基础设施和服务体系建设，进一步取缔集中区外小型非法加工经营企业。推进集中区环境治理，加强原煤入仓加工和煤尘防治，加快集中区绿化。鼓励煤炭企业转产转型，支持开展兼并重组，淘汰实力弱小企业，盘活土地资源。(六)扩大交流合作，全面深入促开放

抢抓“一带一路”建设新机遇，以陆港建设为龙头，加快推进内陆开放型经济试验区建设，积极构建对外开放新格局，努力建设丝绸之路经济带重要节点城市。

着力推动重大专项。认真落实内陆开放三年行动计划，突出重大专项带动，科学评估第一批重大专项实施效果，全面推进十大专项落实。积极争取自治区出台支持石嘴山陆港经济区发展的意见，加快基础设施建设，增强陆港带动能力。组织实施一批道路建设项目，畅通周边通道，提升与周边地区资源共享水平。加快智慧城市建设，引进和整合现有物流信息企业，建立完善物流信息平台和快递物流网络，抓好4G电子商务产业发展。

主动融入中阿博览会。利用中阿博览会平台，进一步深化与阿拉伯国家和穆斯林地区的交流合作。组团赴阿拉伯国家进行友好访问，争取与南非国开普敦市建立友好城市。充分发挥商会、行业协会作用，开展中阿博览会联系国家回访拜会，寻求在能源、矿山机械装备、清真食品、文化旅游等方面的合作。发挥国家级开发区平台优势，深化完善东西部开发区合作发展机制。

深入开展区域合作。推动与农垦集团合作示范园建设，统筹推进旅游资源的保护与开发，共同打造独具特色的旅游目的地。深化与山东、天津及长三角等地的合作，扎实推动宁津物流园和淄山工业园区建设。鼓励企业境外投资，推进盈谷集团在加拿大实施金矿开发。深化与台湾在新材料产业方面合作，加强与台商联系，积极对接一批企业，争取落实一批项目。

(七)发展社会事业，尽心竭力惠民生

按照保基本、守底线的要求，更加重视民生改善和社会建设，促进社会和谐稳定。

全面发展社会事业。深入推进教育领域综合改革，重点实施优质教育资源提质扩面工程，新建示范性综合实践基地，力争在全国率先建成义务教育基本均衡发展城市。深化医药卫生体制改革，加强医疗卫生服务能力建设。加快推进市儿童医院暨妇幼保健院、第一人民医院内科综合楼、第二人民医院全科医生培养基地建设。加强计划生育工作，做好全国婚育新风进万家示范市评估验收。推进国家公共文化体系示范区建设，扩大政府文化资助和文化采购，在标准化、均等化、社会化、数字化方面取得新的突破。引入竞争机制，推动公共文化服务社会化发展，鼓励社会力量、社会资本参与公共文化服务体系建设，培育文化非营利组织。积极备战自治区第八届少数民族传统体育运动会。

提升社会保障水平。推进全民参保登记和社会保险“五险合一”，扩大社会保险覆盖面，推进被征地农民养老保险工作。做好稳定就业工作，积极开展就业帮扶，防止因企业关停和经济性裁员造成失业率急剧攀升。完善大学生创业孵化园功能，加快智能化人力资源市场建设。加大保障性住房建设力度，续建棚户区改造7569户、新建1246户，完成投资16亿元。抓好公共租赁住房建设，新建公共租赁住房2000套，续建563套，新增廉租住房补贴700户，完成投资2.4亿元，建立健全保障性住房长效管理机制。抓好“菜篮子”工程，确保物价总水平基本稳定。健全完善城乡社会救助体系，不断提高灾害预防和救助能力。扎实做好移民安置，认真开展就业培训、产业培育和管理服务等工作。

加强社会治理创新。巩固乡镇(街道)“两个中心”规范化建设和社区网格化管理成果，探索建立民情服务流水线，推进城乡社区自治。健全养老服务业发展政策支撑，完善社区居家养老信息服务平台。强化矛盾纠纷排查，加强特殊人群管理，发挥人民调解作用，有效防范和化解矛盾。严格落实信访责任，加大信访积案清理化解，把涉法、涉诉信访纳入法治渠道解决。推进民族事务法治化，依法管理宗教事务。强化安全生产责任，大力开展重点行业领域专项整治，坚决遏制重特大安全事故发生。严密防范、依法打击各类违法犯罪活动，加大新一轮禁毒人民战争力度，推进平安石嘴山建设。健全应急管理机制，提高应急处置能力。

今年，市政府将继续集中办好十件民生实事，努力让群众满意。1. 加快棚户区改造和公共租赁住房建设，完成棚户区改造安置4000户，公共租赁住房1000套。2. 实施20万平方米老旧住宅小区节能改造；加强物业管理行业指导监管，创建规范化物业服务住宅小区20个。3. 为43所学校安装饮水设备，彻底解决全市农村偏远中小学校师生安全饮水问题。4. 为困难群众免费办理法律援助案件800件。5. 实施全市重度残疾人生活津贴发放全覆盖；为85岁以上老年人发放长寿保健金。6. 实施千人妇女儿童健康保障工程。7. 建设石嘴山市失能失智护理院。8. 新建大学生创业孵化园，扶持引领150名大学生自主创业。9. 扶持全市非营利性社会文化艺术社团组织，提升群众文化活动水平。10. 开展新型职业农民和农村劳动力职业技术培训3000人。

(八)科学编制规划，着眼长远谋发展

突出重点、集思广益，科学编制“十三五”规划，为全市转型发展提供科学指导。

深化重大问题研究。从解决经济社会发展的突出矛盾和问题入手，紧紧围绕自治区“两大战略”、“两区”建设和产业、民生、生态“三大转型”，谋划丝绸之路战略节点城市，重点开展产业优化升级和结构调整、新型城镇化、生态环境保护、基本公共服务和社会保障、创新社会治理、深化改革和扩大开放等方面的研究，为编制好“十三五”规划提供有力支撑。

精心编制发展规划。组织编制国民经济和社会发展“十三五”规划纲要，确定今后一个时期全市的发展目标、发展方向。认真编制重点领域、重点产业发展规划，指导领域发展、生产力布局和政府投资方向。按照县区功能定位，加快各县区总体规划修编。全面总结评价“十二五”规划目标任务落实情况，为规划编制提供科学参考。

加强规划融合衔接。扎实推进“三规合一”试点及“多规融合”工作，将城乡总体规划、土地利用总体规划、国民经济和社会发展“十三五”规划所涉及的空间信息汇集共享，实现产业发展、城乡建设、国土开发等空间信息“一张图”全覆盖，为推动经济社会发展提供科学指导。

建立规划落实体系。健全规划落实的领导体制、工作机制、问责机制和科学考评体系，制定完成规划的路线图与时间表，每年确定工作任务，按期实现工作目标，保证未来五年通过“十三五”规划实施，在转型升级中取得阶段性成果。定期开展规划评估，增强规划的严肃性和时效性，提高规划的执行力，更好发挥规划调控、引领和约束作用。

各位代表，全面完成今年的目标任务，政府肩负着重大而艰巨的责任，承载着全市人民的厚望。我们将把百姓的呼声作为工作指挥棒，把百姓的期望作为工作的风向标，以深化改革为动力，全面加强法治政府、服务政府、廉洁政府建设。

依法行政推进法治政府建设。全面贯彻落实十八届四中全会精神，加快推进依法治市进程，做到依法行政、依法办事、依法维权。全面推行重大决策合法性审查机制，健全完善政府法律顾问团制度。强化对稳增长、促改革等重大政策措施跟踪审计监督，确保政令畅通。深化行政执法体制改革，梳理各部门执法权，着力解决权责交叉、多头执法问题。全面推进政务公开，实现决策、执行、结果公开透明，增强政府公信力。加大干部法治教育培训力度，提高依法行政意识，增强依法履职的能力。积极探索开展立法工作，逐步完善立法工作机制，提高立法的针对性和可操作性。支持法院、检察院依法独立公正行使审判权和检察权，支持执法司法机关推进体制机制改革。扎实开展“六五”普法，深入推进普法依法治理工作，深化法治城市、法治县区、民主法治村(居)等创建活动，推动全社会形成学法、用法、尊法、守法的良好氛围。

深化改革推进服务政府建设。适应新常态的要求，探索建立规划落实体系、政策评估体系、指标约束体系、民生改善体系和考核激励体系，推进政府职能转变。继续深化行政体制改革，建立政府部门权力清单、运行责任清单和审批负面清单“三个清单”制度，为各类市场主体提供公平竞争环境。进一步优化行政审批流程和环节，加强行政审批内部管理和监督，推广行政审批首席代表制度。抓好政务大厅运行，推行并联审批、网上审批，压缩时限、减少程序、提高效率，打造西北服务一流的政务服务中心。推进财政预算管理制度改革，完善财政预算体系，全面推进预决算公开 天火大道无弹窗 。健全完善政府购买公共服务机制，进一步扩大采购预算范围，初步形成政府购买公共服务的平台和机制。稳妥推进公务用车改革，启动机关事业单位养老并轨改革。加强地方政府债务管理，切实防范和化解政府债务风险。

转变作风推进廉洁政府建设。自觉接受人大及其常委会的法律监督、政协的民主监督，广泛接受社会公众和舆论监督。巩固和扩大教育实践活动成果，纠正“四风”和树立新风并举，建立健全作风建设长效机制。以智谋事，以诚促事，以勤成事，始终保持工作热情和创业激情，任务安排在哪里，责任就延伸到哪里，监督就跟进到哪里，落实就推进到哪里，最终成效就在哪里检验。健全完善电视问政、干事档案、季度考评监督机制，强化行政问责和效能监察，大力整治不作为、慢作为、乱作为和庸懒散、生冷硬等问题。严格贯彻落实八项规定精神，加强“三公”经费管理，节约政府开支，倡俭治奢，转变作风。全面落实党风廉政建设主体责任，严守廉洁从政各项规定。健全规范和制约权力运行的制度体系，完善土地出让、产权交易、工程招标、政府采购等公共资源交易体系。加强对重点领域不正之风的专项治理，加大对违法违纪案件的查处力度，真正做到干部清正、政府清廉、政治清明。

各位代表，石嘴山正处在调整结构、转型发展的关键时期，任务艰巨而繁重，责任重大而光荣。让我们在市委的坚强领导下，团结拼搏，迎难而上，以更加振奋的精神，更加扎实的工作，全力推进经济社会持续健康发展，为建设开放富裕和谐美丽石嘴山而努力奋斗!
